# Comparative Efficacy of Conservative Surgery vs Minor Amputation for Diabetic Foot Osteomyelitis

**DOI:** 10.1177/10711007231194046

**Published:** 2023-09-19

**Authors:** Madlaina Schöni, Laura Soldevila-Boixader, Thomas Böni, Javier Muñoz Laguna, Ilker Uçkay, Felix W. A. Waibel

**Affiliations:** 1 Department of Orthopedics, Balgrist University Hospital, University of Zurich, Zurich, Switzerland; 2 Department of Infectious Diseases, Bellvitge University Hospital, Barcelona, Spain; 3 EBPI-UWZH Musculoskeletal Epidemiology Research Group, University of Zurich and Balgrist University Hospital, Zurich, Switzerland; 4 Epidemiology, Biostatistics and Prevention Institute (EBPI), University of Zurich, Zurich, Switzerland; 5 University Spine Centre Zurich (UWZH), Balgrist University Hospital and University of Zurich, Zurich, Switzerland; 6 Department of Infectious Diseases, Balgrist University Hospital, University of Zurich, Zurich, Switzerland

**Keywords:** diabetic foot, osteomyelitis, conservative surgery, comparative effectiveness, amputation

## Abstract

**Background:**

There is uncertainty regarding the optimal surgical intervention for diabetic foot osteomyelitis (DFO). Conservative surgery—amputation-free resection of infected bone and soft tissues—is gaining traction as an alternative to minor amputation. Our primary objective was to explore the comparative effectiveness of conservative surgery and minor amputations in clinical failure risk 1 year after index intervention. We also aimed to explore microbiological recurrence at 1 year, and revision surgery risk over a 10-year study period.

**Methods:**

Retrospective, single-center chart review of DFO patients undergoing either conservative surgery or minor amputation. We used multivariable Cox regression and Kaplan-Meier estimates to explore the effect of surgical intervention on clinical failure (recurrent diabetic foot infection at surgical site within 1 year after index operation), microbiological recurrence at 1 year, and revision surgery risk over a 10-year follow-up period.

**Results:**

651 patients were included (conservative surgery, n = 121; minor amputation, n = 530). Clinical failure occurred in 34 (28%) patients in the conservative surgery group, and in 111 (21%) of the minor amputation group at 1 year (*P* = .09). After controlling for potential confounders, we found no association between conservative surgery and clinical failure at 1 year (adjusted hazard ratio [HR] 1.3, 95% CI 0.8-2.1). We found no between-group differences in microbiological recurrence at 1 year (conservative surgery: 8 [6.6%]; minor amputation: 33 [6.2%]; *P* = .25; adjusted HR 1.1, 95% CI 0.5-2.6). Over the 10-year period, the conservative group underwent significantly more revision surgeries (conservative surgery: 85 [70.2%]; minor amputation: 252 [47.5%]; *P* < .01; adjusted HR 1.3, 95% CI 0.9-1.8).

**Conclusion:**

We found that with comorbidity-based patient selection, conservative surgery in the treatment of DFO was associated with the same rates of clinical failure and microbiological recurrence at 1 year, but with significantly more revision surgeries during follow-up, compared with minor amputations.

**Level of Evidence:**

Level III, retrospective comparative effectiveness study.

## Introduction

Diabetes represents a leading cause of mortality and reduced life expectancy, and its burden is projected to increase.^23^ Among people living with diabetes, it has been estimated that one-third develop a diabetic foot ulcer, and more than one-sixth experience a diabetic foot infection (DFI).^4^ Currently, there is no consensus on the optimal management of DFI and diabetic foot osteomyelitis (DFO), and approximately 20% of patients end up requiring an amputation.^4^ In recent years, alternatives to amputation procedures have been proposed in selected patient subgroups—antibiotic therapy alone^1,2,9,16,25,27^ and conservative surgery (synonyms: internal resection, internal partial foot amputation^24^). The latter involves removal of infected bone and nonviable soft tissue, without amputation.^2,14^

Evidence regarding conservative surgery for DFI and DFO is limited, although they have been applied to forefoot^7,12,15^ as well as mid- and hindfoot cases.^2,3,6,18^ Furthermore, there is marked heterogeneity in the literature with respect to the operational definition of *clinical failure* as well as its ideal time point after conservative surgical interventions for DFO. Yet, clinical failure after conservative surgery has been estimated to range between 0% and 35%.^2,7,18^ Exposed bone, presence of ischemia and necrotizing soft tissue infection,^2^ peripheral arterial disease (PAD), higher C-reactive protein on admission (115.9 ± 112 mg/L in case of failure; 47.6 ± 47.5 mg/L in case of remission) and abscess formation^18^ are factors that have been suggested to be related to clinical failure in conservative surgery.

Despite clinical interest in conservative surgical techniques, they may carry an increased risk of microbiological persistence within exposed residual soft tissue and bone, ultimately limiting their implementation. Research efforts have been made to compare conservative surgeries and minor amputations, with one study finding no between-group differences in terms of complications.^7^ Participants in this study received 4 weeks of antibiotic treatment prior to index surgery. Another study in the Spanish context found similar null results when comparing these 2 surgical approaches for DFO.^3^

We aimed to add to the sparse body of knowledge and explore the effectiveness of a proposed conservative surgery when compared to minor amputation for DFO. Our primary objective was to explore clinical failure 1 year after index intervention (primary outcome). Our secondary objective was to explore microbiological recurrence at 1 year (secondary outcome) and revision surgery risk over a 10-year follow-up period (secondary outcome).

## Patients and Methods

### Reporting

We adhered to applicable items of the Strengthening the Reporting of Observational Studies in Epidemiology (STROBE) and other pertinent methodologic publications for the present work and design.^28,29^

### Setting and Eligibility Criteria

Balgrist University Hospital is a tertiary referral center for orthopaedic surgery running a specialized diabetic foot unit. Our source population consisted of all participants suffering from DFO with available medical records and routinely collected clinical data, treated at our center between 2000 and 2020. We selected surgical group cases and restricted our study population to patient records with sufficient documentation in medical records and follow-up. During this process, we carefully considered the trade-off between external validity and the exploratory objective of this work. We included adult (aged ≥18 years) DFO patients undergoing conservative surgery, or minor amputation interventions, in combination with antibiotics from January 1, 2000, to March 31, 2020. We excluded patients undergoing nonsurgical treatments only, major amputation, and realignment arthrodesis of infected diabetic Charcot feet. The study was approved by the local ethical committee of the canton of Zurich, Switzerland.

### Definitions—Interventions, Outcomes, and Other Relevant Variables

**Figure 1.**
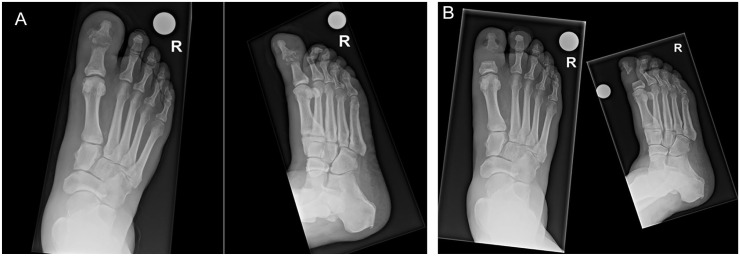
Dorsal-plantar and oblique radiographs of a 69-year-old male patient with osteomyelitis of the first interphalangeal joint (A) before and (B) after conservative surgery.

**Figure 2.**
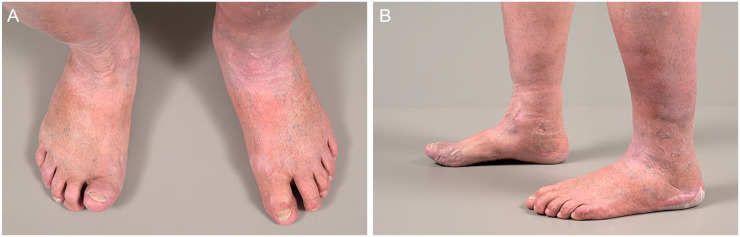
(A and B) Clinical photographs of the patient described above 2 years after conservative surgery of the first interphalangeal joint.

“Conservative surgery” was operationalized as a surgical procedure removing the infected bone and nonviable soft tissue without amputation of any part of the foot.^2,14^ For illustration purposes, Figure 1 presents radiographs before and after conservative surgery of the first interphalangeal joint in a 69-year-old diabetic male suffering from DFO. Similarly, Figure 2 presents bilateral foot photographs of the same patient 2 years after conservative surgery. Amputations between the toes and the ankle joint were considered “minor amputations” in our study.^31^ Both intervention groups received antibiotic treatment after surgery. Duration and choice of postoperative antibiotic treatment followed the most recent IWGDF guidelines.

We defined “clinical failure” as any relevant clinical problem (ie, DFI [including infected ulcers], DFO) occurring within 1 year after index operation at the original surgical site that needed a surgical procedure (surgical debridement in the operation room, conservative, minor or major amputation).^13,30^ Furthermore, we defined “microbiological recurrence” as a clinically recurrent DFI at the same location, with cultures showing at least 50% of the same pathogen as isolated in the index episode.^13,30^ “Revision surgeries” were defined as any new conservative surgery, minor or major amputation due to diabetic foot infections, at any localization within the same foot during the whole follow-up period. Finally, we operationalized “polymicrobial sample” as the presence of 2 or more microorganisms in a culture sample.

### Patient Management and Real-World Clinical Decision Making

DFO was diagnosed by the presence of bacteria in bone samples obtained during conservative surgery or minor amputation and preoperatively using a combination of radiographic examination and magnetic resonance imaging (MRI). If MRI was not possible, we used computed tomography or skeletal scintigraphy. The extent of osteomyelitis was judged by the amount of fat mark extinction in MRI T1 sequences.^5^ Localized osteomyelitis was defined as an osteomyelitis that was restricted to 1 or 2 bones at their articular junction. Soft tissues were clinically assessed on a case-by-case basis, carefully considering whether their viability would allow primary wound closure. PAD was diagnosed using routine angiologic measurements. Severity of PAD, measured with the Fontaine classification,^8^ was not used as a criterion to rule out conservative surgery. Wound healing potential at the surgical site was estimated by our referral angiologists using a combination of TcPO2 measurements, ankle or toe brachial index, and oscillography. The choice of surgical treatment was standardized and made as follows under guidance of a single senior orthopaedic surgeon (TB) with more than 25 years of experience in the treatment of diabetic feet. In general, conservative surgery was favored when all the following criteria were met:

1. Presence of localized as opposed to disseminated osteomyelitis.
2. Viable soft tissues for primary wound closure.
3. Presence of wound healing potential per the referral angiologist’s judgment.
4. Biomechanical suitability.

### Primary and Secondary Study Outcomes

Clinical failure at 1 year was the primary study outcome. Secondary outcomes included microbiological recurrence at 1 year, and revision surgery risk over the 10-year follow-up study period.

### Statistical Analyses

Continuous variables were described as means and SDs or median and interquartile range. Categorical variables were described as numbers and percentages. Baseline characteristics were tested for differences—Student *t* test for continuous variables, and χ^2^ test for categorical variables. Baseline continuous variables severely violating homoskedasticity assumptions were assessed for differences with the Mann-Whitney *U* test. We assessed our primary outcome (clinical failure at 1 year), and secondary outcomes (microbiological recurrence at 1 year, and revision surgery risk over 10 years) using multivariable Cox regression and Kaplan-Meier estimates. Multivariable regression models controlled for key potential confounders—age, sex, peripheral arterial disease, antibiotic treatment prior to index surgery, and anatomical location. These potential confounders were chosen based on clinical expertise and structural assumptions, all within the exploratory objective of this work. We used *time to clinical failure* (primary outcome), *time to microbiological recurrence* (secondary outcome), and *time to revision* (secondary outcome) as dependent variables, surgical intervention as main exposure of interest, and introduced age, sex, peripheral arterial disease, antibiotic treatment prior to index surgery, and anatomical location as covariates in the model. We assessed the proportional hazard assumption in our fitted model by plotting Schoenfeld residuals against time. We then performed a log-rank analysis and plotted the corresponding Kaplan-Meier survival curves for clinical failure-free and microbiological recurrence-free survival for both surgical groups at 1 year and for revision surgery-free survival throughout the 10-year follow-up study period. Analyses were performed using SPSS software (version 28.0; IBM Corp, Armonk, NY) and R (R Foundation for Statistical Computing, Vienna, Austria).^20^

### Sensitivity Analysis

To assess the robustness of the exploratory measures of effect emerging from the multivariable Cox regression models for our primary and secondary outcomes, we opted for performing an alternative analysis using another method for confounding control in observational studies—propensity score matching.^21^ We developed a logistic regression propensity score quantifying the conditional probability of receiving conservative surgery given a vector of key observed covariates—age, sex, peripheral arterial disease, antibiotic treatment prior to index surgery, and anatomical location. We used nearest neighbor as the matching method, and matched (1:1) 121 conservative surgery to 121 minor amputation patients based on the calculated propensity scores and visually explored the distribution of these for each group (see Supplemental Figure S1). We then fit 3 analogous Cox models to the primary analysis but in the matched study population, using *time to clinical failure, time to microbiological recurrence*, and *time to revision* as dependent variables, and surgical intervention as the only independent variable.

## Results

### Characteristics of Study Population

We included 121 DFO patients in the conservative surgery group and 530 patients in the minor amputation group. The 2 surgical groups were comparable in many characteristics. Patients undergoing conservative surgery were, on average, younger, had less coronary heart disease and PAD, had shorter hospital stays and had longer antibiotic therapy postoperatively (Table 1). Supplemental Table 2 presents the characteristics of the propensity score–matched study population.

**Table 1.** Patient demographics.

	Conservative Surgery (n = 121)	Minor Amputation (n = 530)	*P* Value
General characteristics			
Age, y	62 (55-71)	69 (61-76)	**<.01**
Male sex	98 (81)	428 (81)	.95
BMI	28 (25-33)	30 (25-33)	.41
Years of diabetes	18 (9-27)	19 (11-26)	.54
Duration of follow-up, y	3.6 (1.9-6.6)	2.1 (1.0-4.4)	**<.01**
Localization			
Forefoot	28 (23)	275 (52)	**<.01**
Midfoot	74 (61)	233 (44)	
Hindfoot	19 (16)	22 (4)	
Coronary arterial disease	38 (31)	234 (44)	**.01**
Peripheral arterial disease	70 (58)	383 (72)	**.02**
Stage of peripheral arterial disease (Fontaine classification)			.2
Stage I	24 (20)	120 (23)	
Stage II	28 (23)	166 (31)	
Stage III	2 (2)	2 (0.4)	
Stage IV	6 (5)	46 (9)	
Angioplasty	50 (42)	279 (53)	**.03**
Preoperative antibiotic treatment	86 (71)	378 (71)	.96
Duration of preoperative antibiotic treatment, d	14 (6-28)	10 (5-24)	.23
Creatinine value, µmol/L	100 (78-140)	96 (74-128)	.3
GFR	60 (41-86)	62 (44-86)	.6
Preoperative CRP, mg/L	54 (12-162)	14 (8-45)	.13
Days of hospitalization	18 (11-31)	12 (8-24)	**<.01**
Duration of total antibiotic, d	32 (21-48)	19 (14-31)	**<.01**
Negative microbiologic sample	16 (13)	47 (9)	.15
Polymicrobial sample	40 (40)	199 (43)	.6
Clinical failure	34 (28.1)	111 (20.9)	.09
Microbiological recurrence	8 (6.6)	33 (6.2)	.25
Revision	85 (70.2)	252 (47.5)	**<.01**

Abbreviations: BMI, body mass index; CRP, C-reactive protein; GFR, glomerular filtration rate.

Data are shown as n (%) or median (interquartile range). Boldface indicates statistical significance (*P* < .05).

### Clinical Failure

One year after index intervention, clinical failure occurred in 34 (28%) patients in the conservative surgery group, and in 111 (21%) patients in the minor amputation group (*P* = .09). After controlling for the effect of age, sex, peripheral arterial disease, antibiotic treatment prior to index surgery, and polymicrobial osteomyelitis (ie, holding these potential confounders constant), the exploratory risk of clinical failure for the conservative surgery group was not different than in the minor amputation group (hazard ratio [HR] 1.3, 95% CI 0.8-2.1) (Table 2). This main exploratory effect estimate was robust to the method of choice for confounding control and showed similar direction and magnitude among the propensity score–matched study subpopulation, although with less precision (HR 1.5, 95% CI 0.9-2.4; *P* = .16).

**Table 2.** Risk Factors for Clinical Failure.

	Multivariable Analysis, HR (95% CI)	*P* Value
Age	1.0 (0.9-1.0)	.40
Male sex	2.2 (1.1-4.0)	.09
Peripheral arterial disease	0.9 (0.5-1.7)	.80
Polymicrobial sample	1.6 (1.1-2.3)	.02
Preoperative antibiotic therapy	1.4 (0.9-2.1)	.13
Conservative surgery (main exposure of interest)	1.3 (0.8-2.1)	.30

Abbreviation: HR, Hazard Ratio.

**Figure 3.**
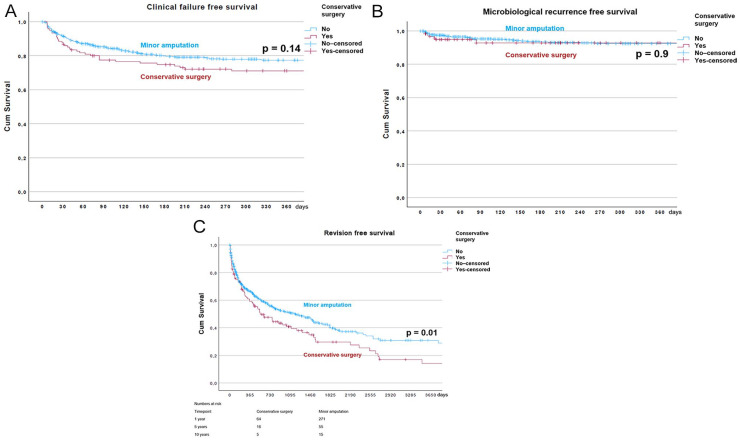
(A) Kaplan-Meier survival estimate for clinical failure–free survival for the groups. (B) Kaplan-Meier survival estimate for microbiological recurrence–free survival for the 2 surgical intervention groups. (C) Kaplan-Meier survival estimate for revision surgery–free survival for the 2 surgical intervention groups.

The Kaplan-Meier survival estimate for clinical failure free survival at 1 year of both surgical intervention groups is shown in Figure 3A.

### Microbiological Recurrence

The number of episodes with microbiological recurrence was not different between the 2 surgical intervention groups (conservative surgery: 8 [6.6%]; minor amputation, n = 33 [6.2%]; *P* = .25) at 1 year. Supplemental Table 1 summarizes the microorganisms found in the index episodes as well as those found in cases of microbiological recurrence. After controlling for potential cofounders, the exploratory risk of microbiological recurrence for the conservative surgery group was not different from among those in the minor amputation group (HR 1.1, 95% CI 0.5-2.6). This secondary exploratory effect estimate was also robust to the method of choice for confounding control (HR among propensity score–matched population 1.0, 95% CI 0.4-2.7; *P* = .97).

The Kaplan-Meier survival estimate for microbiological recurrence–free survival of both surgical intervention groups is presented in Figure 3B.

### Revision Surgeries

The number of revision surgeries over the entire 10-year follow-up study period was significantly higher in the conservative surgery group (conservative surgery: 85 [70.2%]; minor amputation, 252 [47.5%]; *p* ≤ 0.01). After controlling for potential cofounders, the exploratory risk of revision surgeries remained higher in the conservative surgery group, although it failed to reach conventional statistical significance thresholds (HR 1.3, 95% CI 0.9-1.8). Similar risk estimates were found in the propensity score–matched study subpopulation over the 10-year follow-up period, although reaching statistical significance (HR 1.5, 95% CI 1.0-2.0; *P* = .03).

Figure 3C shows the Kaplan-Meier survival estimate for revision-free survival throughout the 10-year follow-up study period for the 2 surgical intervention groups.

## Discussion

In a selected number of DFO patients exposed to either conservative or minor amputation surgery within a musculoskeletal tertiary hospital, the type of surgical intervention was not associated with benefits in terms of clinical failure 1 year after index operation. Although the conservative group showed a marginally higher proportion of clinical failure event rates, these differences did not reach conventional statistical significance thresholds. Microbiological recurrence was also found to be comparable among surgical groups at 1 year. Revision surgeries over the entire 10-year follow-up study period were more frequent in the conservative surgery group.

Given the difference in invasiveness and the potential difference in resources needed for the 2 surgical interventions, our exploratory findings may inform future research aiming to assess the effectiveness of conservative surgery for DFO. Our findings suggest that the potential value of conservative surgery should not be dismissed in clinical practice, especially when soft tissue status and perfusion allow.

DFO patients undergoing conservative surgeries have been previously estimated to reach clinical failure in 0% to 36% of cases.^2,7,18^ Our main clinical failure rates are in line with these estimates and are in close agreement with Nguyen and colleagues^18^—to the best of our knowledge, the most similar study to ours in terms of design. However, it is worth highlighting that our operational definition of clinical failure (ie, any relevant clinical problem [infected ulcer, DFI, or DFO] at the original site occurring within 1 year of index operation) was broad and inclusive compared to alternative stringent definitions. Furthermore, we restricted the period of clinical failure occurrence to 1 year, whereas others have partly stopped patient monitoring after wound healing^2,12^ or 12 weeks after healing,^15^ ultimately creating different censoring scenarios. Apart from the difference in case definitions and time periods, caution should be employed when comparing the findings of our exploratory study with those from studies involving patients treated with 4 weeks of antibiotic therapy prior to surgical therapy.^7^ Furthermore, the duration of antibiotic treatment after surgery would benefit from more high-quality research aiming to promote antibiotic stewardship.^11^

We explored a main exposure of interest (surgical intervention) and selected a set of key potential confounders in our study. Yet, we were unable to confirm previously described risk factors—presence of ischemia,^2^ peripheral arterial disease, and higher C-reactive protein on admission.^18^ This may be related to both clinical and methodologic heterogeneity, notable in DFO,^26^ which limits direct comparison between study findings.

In our selected sample, microbiological recurrence was comparable between the 2 surgical intervention groups. This is consistent with previous work.^10,17,19,22^ Despite our exploratory aim, this finding may contest the idea that microorganisms persist more often in conservative surgeries.

Revision surgeries were more frequent in the conservative surgery group over the entire 10-year follow-up study period. Although they should be interpreted with caution, our revision estimates challenge those of Aragón-Sánchez et al,^3^ who found no difference in new amputations between conservative and minor amputation surgeries in a smaller DFO study population (n = 108). These contradictory results may be due, in part, to different operational definitions of “revision” as well as noncomparable interventions, and study populations. Overall, surgeons should foster shared decision making and inform their DFO patients of the mid- to long-term revision risk associated with conservative surgery.

### Limitations

Our retrospective chart review study is not exempt from limitations. First, our pragmatic selection of surgical participants did not foster external validity. However, routinely collected data have inherent challenges with respect to their use for research purposes, and generalizability may not be prioritized. Second, as in any observational study, residual confounding cannot be ruled out, especially with respect to extreme cases of confounding by indication, as clinicians may systematically show a clear preference for one surgical intervention over another when certain comorbidities are present in DFO (ie, peripheral arterial disease). Third, we did not preregister the protocol for the present study, leading to some post hoc decisions regarding the statistical analysis.

## Conclusion

In the treatment of diabetic foot osteomyelitis, we found that with careful patient selection (presence only of localized osteomyelitis, viable soft tissue envelope, competent vascularization, and biomechanical stability) conservative surgery can be used with the same amount of clinical failure and microbiological recurrence at 1 year. However, we did also find that with our conservative surgery approach patients required significantly more revision surgeries compared to minor amputations.

## Supplemental Material

sj-docx-2-fai-10.1177_10711007231194046 – Supplemental material for Comparative Efficacy of Conservative Surgery vs Minor Amputation for Diabetic Foot Osteomyelitis Click here for additional data file. Supplemental material, sj-docx-2-fai-10.1177_10711007231194046 for Comparative Efficacy of Conservative Surgery vs Minor Amputation for Diabetic Foot Osteomyelitis by Madlaina Schöni, Laura Soldevila-Boixader, Thomas Böni, Javier Muñoz Laguna, Ilker Uçkay and Felix W. A. Waibel in Foot & Ankle International

sj-docx-3-fai-10.1177_10711007231194046 – Supplemental material for Comparative Efficacy of Conservative Surgery vs Minor Amputation for Diabetic Foot Osteomyelitis Click here for additional data file. Supplemental material, sj-docx-3-fai-10.1177_10711007231194046 for Comparative Efficacy of Conservative Surgery vs Minor Amputation for Diabetic Foot Osteomyelitis by Madlaina Schöni, Laura Soldevila-Boixader, Thomas Böni, Javier Muñoz Laguna, Ilker Uçkay and Felix W. A. Waibel in Foot & Ankle International

sj-pdf-1-fai-10.1177_10711007231194046 – Supplemental material for Comparative Efficacy of Conservative Surgery vs Minor Amputation for Diabetic Foot Osteomyelitis Click here for additional data file. Supplemental material, sj-pdf-1-fai-10.1177_10711007231194046 for Comparative Efficacy of Conservative Surgery vs Minor Amputation for Diabetic Foot Osteomyelitis by Madlaina Schöni, Laura Soldevila-Boixader, Thomas Böni, Javier Muñoz Laguna, Ilker Uçkay and Felix W. A. Waibel in Foot & Ankle International

sj-png-4-fai-10.1177_10711007231194046 – Supplemental material for Comparative Efficacy of Conservative Surgery vs Minor Amputation for Diabetic Foot Osteomyelitis Click here for additional data file. Supplemental material, sj-png-4-fai-10.1177_10711007231194046 for Comparative Efficacy of Conservative Surgery vs Minor Amputation for Diabetic Foot Osteomyelitis by Madlaina Schöni, Laura Soldevila-Boixader, Thomas Böni, Javier Muñoz Laguna, Ilker Uçkay and Felix W. A. Waibel in Foot & Ankle International
