# Supplementary material for: Comparative Efficacy of Conservative Surgery vs Minor Amputation for Diabetic Foot Osteomyelitis
Source: Foot Ankle Int. 2023 Sep 19;44(11):1142–9. doi: 10.1177/10711007231194046 (PMC10666512; doi:10.1177/10711007231194046)
Supplement: sj-docx-2-fai-10.1177_10711007231194046 – Supplemental material for Comparative Efficacy of Conservative Surgery vs Minor Amputation for Diabetic Foot Osteomyelitis [file sj-docx-2-fai-10.1177_10711007231194046.docx]

| **Minor amputation** | | | |
| --- | --- | --- | --- |
| **First episode** | | | **Microbiological failure episode** |
| **pathogen1** | **pathogen2** | **pathogen3** | **Pathogens** |
| MR-*S.epidermidis* | *Prevotella spp* | . | *Prevotella spp* |
| *S.aureus* | . | . | *S.aureus* |
| MR-*S.epidermidis* | *Enterococcus spp* | . | MR-*S.epidermidis* |
| *Anaerococcus spp* | *E.cloacae* | *E.faecalis* | *Anaerococcus spp, E.faecalis, Bacteroides fragilis* |
| *Enterococcus spp* | *Helococcus kunzii* | *.* | NCS*, Enterococcus spp,*  *Morganella morgagnii* |
| *S.aureus* | *.* | *.* | *S.aureus* |
| *E.faecalis* | *.* | *.* | *E.faecalis* |
| *E.coli* | *Citrobacter freundii* | *S.caprae/capitis* | Polymicrobial |
| *S.haemolyticus* | *Brevundimonas diminuta* | . | MR-*S.epidermidis* |
| Multiresistant *S.epidermidis* | *S.epidermidis* | *Anaerococcus spp* | MR- *S.epidermidis*,  *Prevotella spp* |
| *Proteus vulgaris* | *Enterococcus spp* | *Bacteroides fragilis* | *Proteus vulgaris*, *Bacteroides spp* |
| *S.aureus* | *.* | *.* | *S.aureus* |
| *S.aureus* | *.* | *.* | *S.aureus* |
| Polymicrobial | . | . | Polymicrobial |
| *S.epidermidis* | *.* | *.* | *S.epidermidis* |
| *Enterococcus spp* | NCS | *Morganella morganii* | Polymicrobial |
| MR-*S.epidermidis* | *Prevotella spp* | . | MR-*S.epidermidis*,  *Prevotella spp* |
| *E.coli* | NCS | . | NCS |
| Polymicrobial | . | . | Polymicrobial |
| anaerobe flora mixta | . | . | anaerobes |
| *E.cloacae* | *E.faecalis* | NCS | *S.caprae/capitis,S.epidermidis* |
| *E.cloacae* | NCS | . | Polymicrobial |
| *Meticillin resistant S.aureus* | . | *.* | *S.aureus* |
| MR-*S.epidermidis* | *Prevotella bivia* | . | MR*-S.epidermidis, Prevotella spp* |
| Anaerococcus spp | . | . | Anaerococcus spp |
| *Pseudomonas aeruginosa* | *S.aureus* | *S.epidermidis* | Polymicrobial |
| Polymicrobial | . | . | Polymicrobial |
| **Conservative Surgery** | | | |
| **First episode** | | | **Microbiological failure episode** |
|  |  |  |  |
| Polymicrobial | . | . | Polymicrobial |
| NCS | . | . | NCS |
| NCS | *E.faecalis* | . | NCS, E.faecalis |
| Polymicrobial | *.* | . | Polymicrobial |
| *E.cloacae* | *.* | *.* | *E.cloacae* |
| *K.pneumoniae* | *E.faecalis* | . | Polymicrobial |
| Polymicrobial | . | . | Polymicrobial |
| *S.aureus* | MR-*S.epidermidis* | . | Polymicrobial |

Footnote: MR-S.epidermidis, Multiresistant Staphylococcus epidermidis; spp, species; *S.aureus*, *Staphylococcus aureus*; NCS, negative coagulase staphylococci; *E.faecalis, Enterococcus faecalis; E.coli, Escherichia coli; E. cloacae, Enterobacter cloacae; Polymicrobial, more than 1 microorganism and 50% of identical microorganism from the first episode.*
