# Supplementary material for: Comparative Efficacy of Conservative Surgery vs Minor Amputation for Diabetic Foot Osteomyelitis
Source: Foot Ankle Int. 2023 Sep 19;44(11):1142–9. doi: 10.1177/10711007231194046 (PMC10666512; doi:10.1177/10711007231194046)
Supplement: sj-docx-3-fai-10.1177_10711007231194046 – Supplemental material for Comparative Efficacy of Conservative Surgery vs Minor Amputation for Diabetic Foot Osteomyelitis [file sj-docx-3-fai-10.1177_10711007231194046.docx]

Supplemental Table 2: Patient demographics of the propensity score matched population.

|  | | Minor Amputation  (n = 121) | | | Conservative surgery  (n = 121) | | |  |
| --- | --- | --- | --- | --- | --- | --- | --- | --- |
| Characteristic | |  | | |  | | |  |
| Age—mean (SD) | | 62.11 (12.97) | | 62.21 (12.82) | | |  |  |
| Sex, No. (%) | |  | |  | | | |  |
| Female | 21 (17.4) | 23 (19.0) | | |  |  |  |  |
| Male | 100 (82.6) | | 98 (81.0) | | | |  |  |
| Peripheral arterial disease, No. (%) | | 72 (59.5) | | 70 (57.9) | | | |  |
| Preoperative antibiotic use, No. (%) | | 90 (74.4) | | 86 (71.1) | | | |  |
| Anatomical location | | | | | | | | |
| Forefoot | 103 (85.1) | | 95 (78.5) | | | |  |  |
| Midfoot | 13 (10.7) | | 8 (6.6) | | | |  |  |
| Hindfoot | 5 (4.1) | | 18 (14.9) | | | |  |  |
