# Supplementary figures and images for: Comparative Efficacy of Conservative Surgery vs Minor Amputation for Diabetic Foot Osteomyelitis
Source: Foot Ankle Int. 2023 Sep 19;44(11):1142–9. doi: 10.1177/10711007231194046 (PMC10666512; doi:10.1177/10711007231194046)

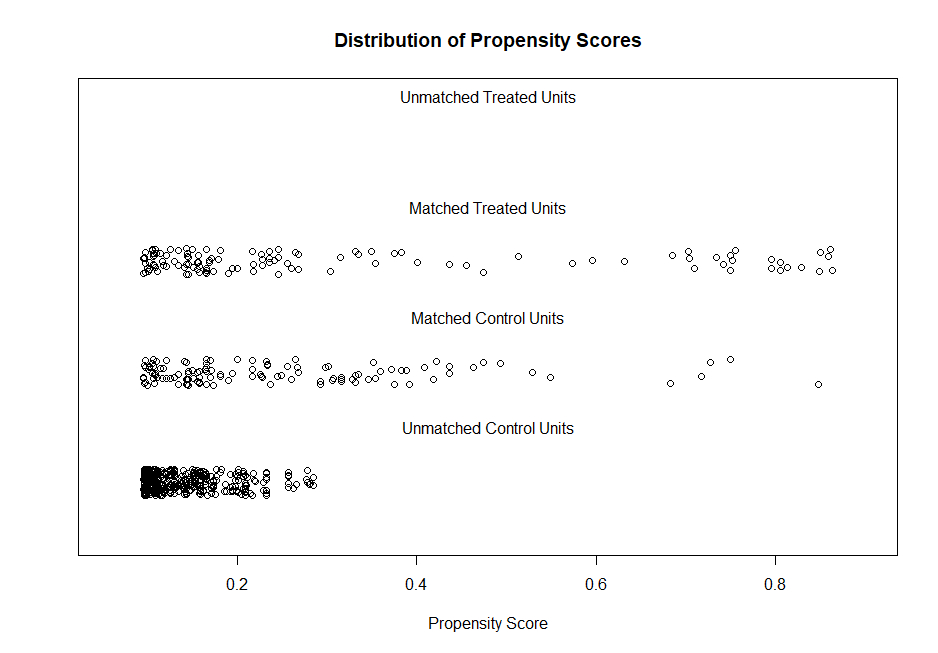

Supplement: sj-png-4-fai-10.1177_10711007231194046 – Supplemental material for Comparative Efficacy of Conservative Surgery vs Minor Amputation for Diabetic Foot Osteomyelitis [file sj-png-4-fai-10.1177_10711007231194046.png]
